# Bio-valorization of *Caragana korshinskii* forage via a synthetic microbial community

**DOI:** 10.1186/s12866-026-05353-5

**Published:** 2026-07-10

**Authors:** Liying Liu, Maoxing Fu, Jiuzhou Peng, Ting Duan, Xiangdong Ma, Hongmei Liu, Rentuya Sha, Yiwen Yang, Hui Yan, Ruiting Jia, Xing Li, Xilong An, Yinghao Liu, Qiang Lu

**Affiliations:** 1 Inner Mongolla Academy of Forestry Sclences, Hohhot, 010010 China; 2 School of Forestry and Grassland Science, Ningxia University, Yinchuan, 750021 China; 3 Inner Mongolia Pratacultural Technology Innovatian Center Co. Ltd, Hohhot, China; 4 Inner Mongolia Autonomous Region Agricultural and Livestock Products Marketing and Sales Service Center, Hohhot, China; 5 Grassland Research Institute, Chinese Academy of Agricultural Sciences, Hohhot, 010010 China

**Keywords:** *Caragana korshinskii*, Silage fermentation, *Bacillus subtilis*, Complex fermentation flora, Microbial community structure

## Abstract

*Caragana korshinskii* represents a critical ecological and feed resource in arid regions, yet its utilization is severely impeded by the recalcitrant lignocellulose barrier. This study established a cross-kingdom synthetic microbial community (SynCom) to synergistically overcome this bottleneck, integrating *Lactobacillus plantarum* for rapid acidification with the fibrolytic enzyme secretion of *Bacillus subtilis* and the oxidative delignification potential of *Aspergillus niger.* We integrated microbiome profiling and functional prediction to decode the fermentation dynamics and metabolic mechanisms. Results demonstrated that the SynCom (LBA) treatment engineered a robust fermentation system, achieving a significantly higher in vitro dry matter digestibility (49.68%) and neutral detergent fiber digestibility (25.65%) compared to the control (*P* < 0.05). This enhancement was driven by a directed shift in the microbiome, where *Lactobacillus* abundance surged to > 95%, effectively suppressing spoilage genera like *Staphylococcus* and *Weissella* via competitive exclusion. Metagenomic prediction revealed that the SynCom upregulated key metabolic modules, specifically pyruvate metabolism and amino acid biosynthesis pathways, facilitating rapid acidification and protein preservation. These findings delineate a coordinated degradation-fermentation-preservation process driven by a rationally assembled synthetic consortium, offering a promising and sustainable bio-valorization strategy for converting high-fiber woody biomass into high-quality livestock feed.

**Supplementary Information:**

The online version contains supplementary material available at https://doi.org/10.1186/s12866-026-05353-5.

## Background

*Caragana korshinskii* stands as a pivotal perennial shrub resource in arid and semi-arid regions, characterized by exceptional ecological adaptability and regenerative potential [1]. As a representative non-grain woody forage, *C. korshinskii* offers substantial biomass and a rich nutritional profile, containing 13–15% crude protein on a dry matter basis alongside diverse bioactive secondary metabolites, particularly amino acids, flavonoids, and terpenoids [2]. Previous investigations indicate that these constituents significantly ameliorate rumen fermentation parameters, optimize nitrogen utilization, and bolster antioxidant capacity by reshaping the rumen microbiome (e.g., elevating Firmicutes abundance to 90%) and activating metabolic pathways (e.g., amino acid biosynthesis) [3], while also modulating immune function in livestock [4]. Consequently, these dual nutritional and functional attributes position it as a strategic feed resource for advancing ecological animal husbandry in desertified regions, possessing profound economic and ecological significance.

However, the primary bottleneck restricting the efficient valorization of *C. korshinskii* biomass lies in its recalcitrant cell wall architecture. The plant possesses a complex lignocellulosic matrix composed of tightly cross-linked polymers that form a formidable physical barrier against microbial degradation [5]. This structural complexity not only restricts substrate accessibility but also impedes the enzymatic hydrolysis required for nutrient release [6, 7]. Consequently, direct feeding of unprocessed material results in suboptimal digestibility, thereby limiting its nutritive value for ruminants. Although physicochemical pretreatments have been explored to mitigate these limitations, they are often associated with high energy consumption and environmental pollution risks. Therefore, the development of efficient, sustainable, and eco-friendly biotechnological strategies to enhance the degradation and conversion of *C. korshinskii* resources remains a priority in current feed science research.

To facilitate the fermentative bioconversion of *C. korshinskii* silage, microbial inoculation is widely recognized as a viable and green strategy. Prior screening studies have established that *Bacillus subtilis* exhibits robust extracellular enzyme secretion capable of modifying rigid plant structures, whereas *Lactobacillus plantarum* ensures rapid acidification during ensiling, effectively suppressing spoilage pathogens and stabilizing the fermentation matrix [8, 9]. Additionally, *Aspergillus niger* is distinguished by its ability to produce a diverse array of oxidative enzymes that further depolymerize complex polysaccharides and enhance substrate porosity [10]. Nevertheless, existing interventions have predominantly relied on single-strain inoculants, which often demonstrate functional limitations in practical applications. Individual strains frequently suffer from poor colonization or metabolic insufficiency due to the dynamic and competitive nature of the silage environment, leading to unstable fermentation outcomes. These constraints underscore that no single microorganism can simultaneously orchestrate rapid acidification, deep structural modification, and long-term stability. Consequently, a critical scientific challenge is to integrate complementary microbial functions via interspecific interactions to construct a coordinated and resilient fermentation system. This has catalyzed increasing interest in synthetic microbial communities (SynComs) as a promising framework to achieve functional synergy and optimize the overall quality of *C. korshinskii* silage.

In recent years, synergistic degradation strategies utilizing compound microbial agents have garnered significant attention. However, systematic insights into the mechanisms of microbiota interactions and their subsequent regulation of metabolic networks remain limited, particularly regarding the specific role of KEGG functional modules during the degradation of lignocellulosic silage [11]. Addressing this knowledge gap, this study innovatively proposes a “targeted regulation of functional flora” strategy by constructing a synthetic consortium comprising *L. plantarum*, *B. subtilis*, and *A. niger*. By employing metagenomic approaches, we systematically explored the synergistic mechanisms within this *Lactobacillus-Bacillus-Fungi* cross-kingdom symbiotic system and their collaborative effects on *C. korshinskii* silage. The specific objective was to elucidate microbial metabolic patterns by decoding the regulatory mechanisms of key pathways, such as pyruvate metabolism and benzoate degradation, and to assess the subsequent improvement in in vitro rumen fermentation characteristics. Ultimately, this work establishes an integrated “degradation-fermentation-nutrient preservation” process for *C. korshinskii* silage, providing a verifiable technical model for the bio-valorization of woody forage resources and offering a theoretical foundation for the development of next-generation microbial additives for sustainable animal husbandry in desert ecosystems.

## Materials and methods

### Substrates and microbial additives

The experiment was conducted using *Caragana korshinskii* (Kom.) harvested from a cultivated plantation in Yanchi County, Ningxia, China (107°E, 37°N) (*Caragana korshinskii* samples were collected with prior permission from the land owner, and all collection procedures complied with relevant institutional and local regulations.). Whole plants (3–4 years old) were harvested at a stubble height of 10 cm on June 30, 2023. To break down the lignified cell walls and promote fermentation, the fresh shrub materials are cut after harvest and ground into powder using an RC-800 grinder until all the materials pass through the sieve. Finally, uniform fine particles with a size of 1–2 millimeters are obtained.

The inoculants used in this study included two bacterial strains, *Bacillus subtilis* (Accession No. OR591286) and *Lactobacillus plantarum* (Accession No. OR591302), and one fungal strain, *Aspergillus niger* (ATCC 16404). The bacterial strains were isolated and screened in our previous study for their high enzymatic and acidifying activities, respectively [12].

### Experimental design and silage processing

A single-factor experimental design was employed with four treatments: (1) CK, control treated with an equivalent volume of sterile distilled water; (2) LB, inoculated with *L. plantarum*; (3) BS, inoculated with *B. subtilis*; and (4) LBA, inoculated with a composite microbial consortium of *L. plantarum*, *B. subtilis*, and *A. niger*. Each treatment was prepared in three independent biological replicates.

Prior to ensiling, fresh *Caragana korshinskii* material was chopped using a grinder to facilitate microbial access and fermentation efficiency, and the processed substrate was adjusted to a moisture content of approximately 50%–55% with sterile water. For the microbial treatments, inoculants were evenly applied to achieve a final density of 1.0 × 10^8 CFU/g fresh weight (FW). The CFU values were estimated based on optical density at 600 nm (OD600) of inoculum cultures during the exponential growth phase. For the LBA group, the three strains were mixed at an equal ratio (1:1:1) prior to inoculation. After thorough mixing, the materials were packed into polyethylene vacuum-sealed silage bags, and the integrity of the seal was checked to ensure anaerobic storage. The bags were then fermented for 30 days at ambient temperature (23–28 °C).

### Analysis of chemical composition and fermentation quality

Take 20 g of the raw material sample and homogenize it for 2 min in a Stomacher type homogenizer using 180 milliliters of sterile distilled water. The mixture was filtered through four layers of medical gauze and qualitative filter paper to obtain the silage extract for pH and organic acid analysis.

The remaining fresh samples were deactivated in an oven at 105℃ for 30 min and subsequently dried to a constant weight for dry matter (DM) determination. The dried samples were ground to pass through a 1-mm screen (60-mesh) for chemical analysis. Crude protein (CP) was determined via the Kjeldahl method. Water-soluble carbohydrates (WSC) were extracted and quantified according to the protocols of AOAC [13] and Owens et al. [14]. Specifically, the WSC content was determined using the anthrone-sulfuric acid colorimetric method [15]. Structural carbohydrates, including neutral detergent fiber (NDF), acid detergent fiber (ADF), and acid detergent lignin (ADL), were analyzed using an ANKOM 2000i Fiber Analyzer (ANKOM Technology, Macedon, NY, USA). The ADL content was determined following the method described by Van Soest et al. [16] to estimate cellulose, hemicellulose, and lignin fractions.

### In vitro digestibility and gas production

Rumen fluid was collected from three fistulated Tan sheep (Tianyuan Sheep Farm, Hongshibao, China) fed a standard maintenance diet. The fluid was strained through four layers of cheesecloth and transported to the laboratory in pre-warmed thermos flasks under anaerobic conditions.

In vitro dry matter digestibility (IVDMD) and neutral detergent fiber digestibility (IVNDFD) were determined using the two-stage technique described by Tilley and Terry [17]. Briefly, dried samples were incubated with buffered rumen fluid (buffer: rumen fluid ratio = 2:1) at for 48 h, followed by digestion with acid-pepsin solution for another 48 h.

In vitro gas production was measured using the ANKOM RFS Gas Production System (ANKOM Technology, USA). A 1.0 g sample was incubated with 110 mL of buffered rumen fluid in 250 mL glass modules for 48 h. Cumulative gas production was recorded automatically, and blank controls were included to correct for gas produced by the rumen fluid alone.

### Microbial DNA extraction and sequencing

For microbial community analysis, 10 g of 30-day fermented silage samples and fresh raw material were mixed with 90 mL sterile saline and agitated to detach microorganisms. The suspension was filtered through sterile gauze and centrifuged at 12,000 rpm 4 ℃ for 15 min to harvest the microbial pellet. Total genomic DNA was extracted using the E.Z.N.A.^®^ Soil DNA Kit (Omega Bio-tek, Norcross, GA, USA) according to the manufacturer’s protocol.

The V3–V4 hypervariable region of the bacterial 16S rRNA gene was amplified using primers 338F (5’-ACTCCTACGGGAGGCAGCAG-3’) and 806R (5’-GGACTACHVGGGTWTCTAAT-3’). Sequencing was performed on an Illumina NextSeq 2000 platform, and data processing was carried out as previously described [18]. Operational taxonomic units (OTUs) were clustered at 97% similarity using UPARSE software (version 7.1) [19], with singletons and chimeras removed. Taxonomic classification was performed using the RDP Classifier against the Silva 138 database with a confidence threshold of 70% [20]. Functional profiles of bacterial communities were predicted using Tax4Fun based on 16 S rRNA gene sequencing data [21].

To complement the 16 S rRNA gene-based analysis and directly assess the genetic potential of the entire microbial community, shotgun metagenomic sequencing was performed on selected silage samples. Total genomic DNA was extracted using the Mag-Bind^®^ Soil DNA Kit (Omega Bio-tek, Norcross, GA, USA) and quality-checked by fluorometry, spectrophotometry, and agarose gel electrophoresis. DNA was fragmented to ~ 350 bp and used to construct paired-end libraries with the NEXTFLEX Rapid DNA-Seq Kit (Bioo Scientific). Sequencing was performed on an Illumina NovaSeq 6000 platform (Illumina Inc., San Diego, CA, USA) at Majorbio Bio-Pharm Technology Co., Ltd. (Shanghai, China).

### Statistical and ecological analysis

Data regarding fermentation quality and alpha diversity were analyzed by one-way analysis of variance (ANOVA) using SPSS 27.0. Significant differences among means were determined using Tukey’s HSD test (*P* < 0.05). To decipher the community assembly mechanisms, a Neutral Community Model (NCM) was applied to quantify the importance of stochastic processes. These ecological analyses were performed using the Majorbio Cloud Platform. For the Spearman correlation analyses between microbial taxa and silage fermentation parameters, the reported P values were not adjusted for multiple comparisons because these analyses were exploratory. Thus, the correlations should be interpreted as preliminary findings requiring further validation.

## Results

### Nutritional ingredients of raw materials

Based on the data shown in Table 1, the *Caragana* raw material has a DM content of 652.42 g/kg, a CP content of 135.00 g/kg, and a WSC content of 32.04 g/kg. The contents of NDF, ADF, and ADL are 678.91 g/kg, 486.21 g/kg, and 225.18 g/kg, respectively.

**Table 1:** Nutritional ingredients of *Caragana korshinskii*

Items	Caragana korshinskii
dry matter DM (g/kg FM)	652.42
crude protein CP (g/kg DM)	135.00
water soluble carbohydrates WSC (g/kg DM)	32.04
neutral detergent fiber NDF (g/kg DM)	678.91
acid detergent fiber ADF (g/kg DM)	486.21
Acid lignin ADL (g/kg DM)	119.86
Cellulose (g/kg DM)	366.35
Hemicellulose (g/kg DM)	192.70

*FM* Fresh material basis, *DM* Dry matter basis, *CP* Crude protein, *WSC* Water-soluble carbohydrate, *NDF* Neutral detergent fiber, *ADF* Acid detergent fiber, *ADL* Acid lignin

### Effects of compound fermentation bacteria on in vitro rumen fermentation characteristics of *Caragana korshinskii* silage

The in vitro dry matter digestibility and in vitro neutral detergent fiber digestibility of *Caragana korshinskii* silage at different stages are shown in Table 2. The IVDMD and IVNDFD of all treatment groups were significantly different (*P* < 0.05), and the addition of *Lactobacillus plantarum*, *Bacillus subtilis* and *Aspergillus niger* could significantly improve IVDMD and IVNDFD. At 24 h of in vitro fermentation, IVDMD and IVNDFD in CK treatment group were significantly lower than those in other treatment groups (*P* < 0.05). The LBA treatment group showed significantly higher IVDMD and IVNDFD compared to the other treatment groups at 30 d of ensiling, reaching 35.05% and 17.05%, respectively (*P* < 0.05). Compared to 7 d of ensiling, its IVDMD increased by 4.04%. At 7 d, 14 d, and 30 d of ensiling, no significant differences in IVNDFD were observed among the treatment groups, except for the CK group.

**Table 2:** Effects of different fermentation periods on digestion of Caragana korshinskii silage in vitro

Incubation Time	Items	Treatment	Ensiling days			SEM	*p*-value		
			7 d	14 d	30 d		T	D	T×D
24 h	IVDMD	LBA	31.01Ba	33.62Aa	35.05Aa	0.18	< 0.01	< 0.01	0.25
		LB	30.67Ba	32.65Aa	32.15ABb				
		BS	31.43Aa	32.47Aa	32.83Ab				
		CK	26.15Bb	27.56ABb	29.46Ac				
	IVNDFD	LBA	14.66Aa	16.13Aa	17.05Aa	0.21	0.35	< 0.01	0.35
		LB	15.37Aa	15.13Ab	15.63Aab				
		BS	15.81Aa	14.64Ab	14.90Abc				
		CK	13.84Aa	12.73Bc	14.00Ac				
48 h	IVDMD	LBA	48.90Aa	49.11Aab	49.68Aa	0.22	0.06	< 0.01	0.07
		LB	45.54Ab	46.81Abc	47.36Aab				
		BS	46.64Bab	50.32Aa	46.35Bbc				
		CK	45.29Ab	45.52Ac	44.83Ac				
	IVNDFD	LBA	28.59Aa	27.89Aa	29.40Aa	0.30	0.04	< 0.01	0.18
		LB	23.57Ab	28.42Aa	27.93Aa				
		BS	25.45Aab	26.94Aa	26.07Ab				
		CK	23.83Bb	24.16Ba	25.65Ab				

Values are presented as means (*n* = 3). Different lowercase superscripts (a-c) within the same column indicate significant differences among treatments at a specific ensiling day (*P* < 0.05). Different uppercase superscripts (A-C) within the same row indicate significant differences among ensiling days for a specific treatment (*P* < 0.05)

*CK* Control group, *LBA* Mixed group of *Lactobacillus plantarum*, *Bacillus subtilis* and *Aspergillus niger*, *LB Lactobacillus plantarum*, *BS Bacillus subtilis* group, *SEM* Standard error of the mean, *T* Treatment effect, *D* Ensiling day effect, T×D, interaction effect

After 48 h of in vitro fermentation, at 30 d of ensiling, the IVNDFD of the CK treatment group was 25.65%, significantly lower than that of the other treatment groups (*P* < 0.05). No significant difference in IVNDFD was observed between the LBA and LB treatment groups at 30 d of ensiling. The effects of microbial inoculation on IVDMD were dependent on the ensiling stage. At 14 d of ensiling, the BS treatment showed the highest IVDMD (50.32%), significantly higher than that of the other treatment groups (*P* < 0.05), whereas at 30 d of ensiling, the LBA treatment exhibited the highest IVDMD (49.68%) (*P* < 0.05). These results indicate that the relative performance of the treatments varied across fermentation stages rather than showing a single treatment that consistently outperformed all others throughout the entire ensiling process.

### Effects of different treatments on in vitro gas production of *Caragana korshinskii* silage

**Fig. 1:**
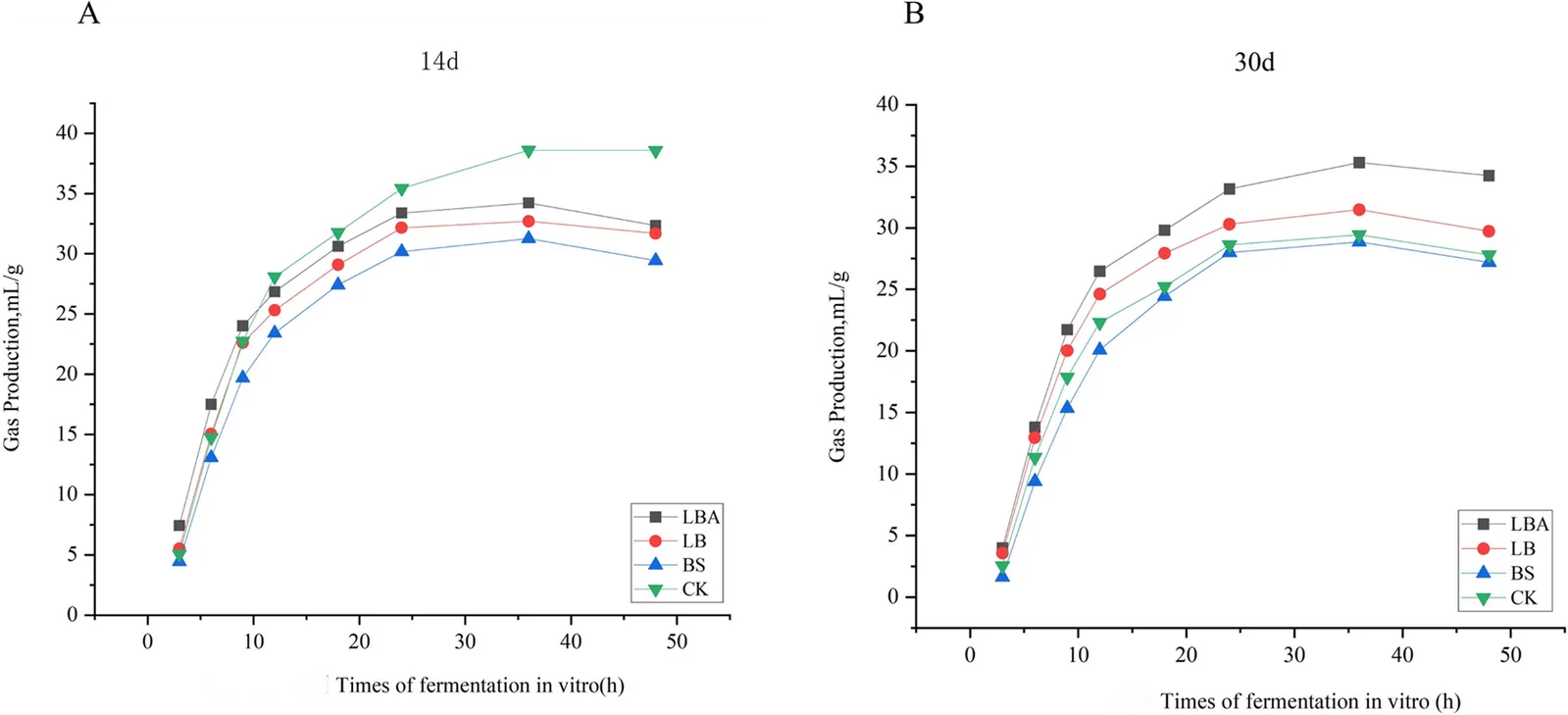
Effect of Composite Fermentation Microflora on In Vitro Gas Production in Caragana korshinskii Silage. **A** Gas production after 14 days of fermentation. **B** Gas production after 30 days of fermentation. LBA, (*L. plantarum* + *B. subtilis* + *A. niger*), LB, (*L. plantarum*), BS, (*B. subtilis*), and CK, (control)

In vitro gas production was evaluated separately for silage sampled after 14 and 30 days of ensiling (Fig. 1). For the 14-day silage, cumulative gas production did not differ significantly among treatments during the early incubation period from 3 to 24 h (*P* > 0.05). After 12 h, the CK group showed relatively higher gas production than the inoculated groups. At 36 h, the LBA and LB treatments showed significantly higher gas production than the BS treatment (*P* < 0.05), and at 48 h, the BS treatment remained significantly lower than the other treatments (*P* < 0.05). For the 30-day silage, treatment differences became clearer. At 6 h of in vitro incubation, the LBA treatment showed significantly higher gas production than the other groups (*P* < 0.05). During the 6–12 h interval, the BS treatment consistently showed the lowest gas production (*P* < 0.05).

Taken together, these results indicate that treatment effects on gas production depended on both ensiling stage and in vitro incubation time. The advantage of the synthetic consortium (LBA) became more evident after 30 days of ensiling, while the BS treatment tended to show lower fermentability across several incubation points.

### Effect of compound fermentation flora on microorganism of *Caragana korshinskii* silage

**Fig. 2:**
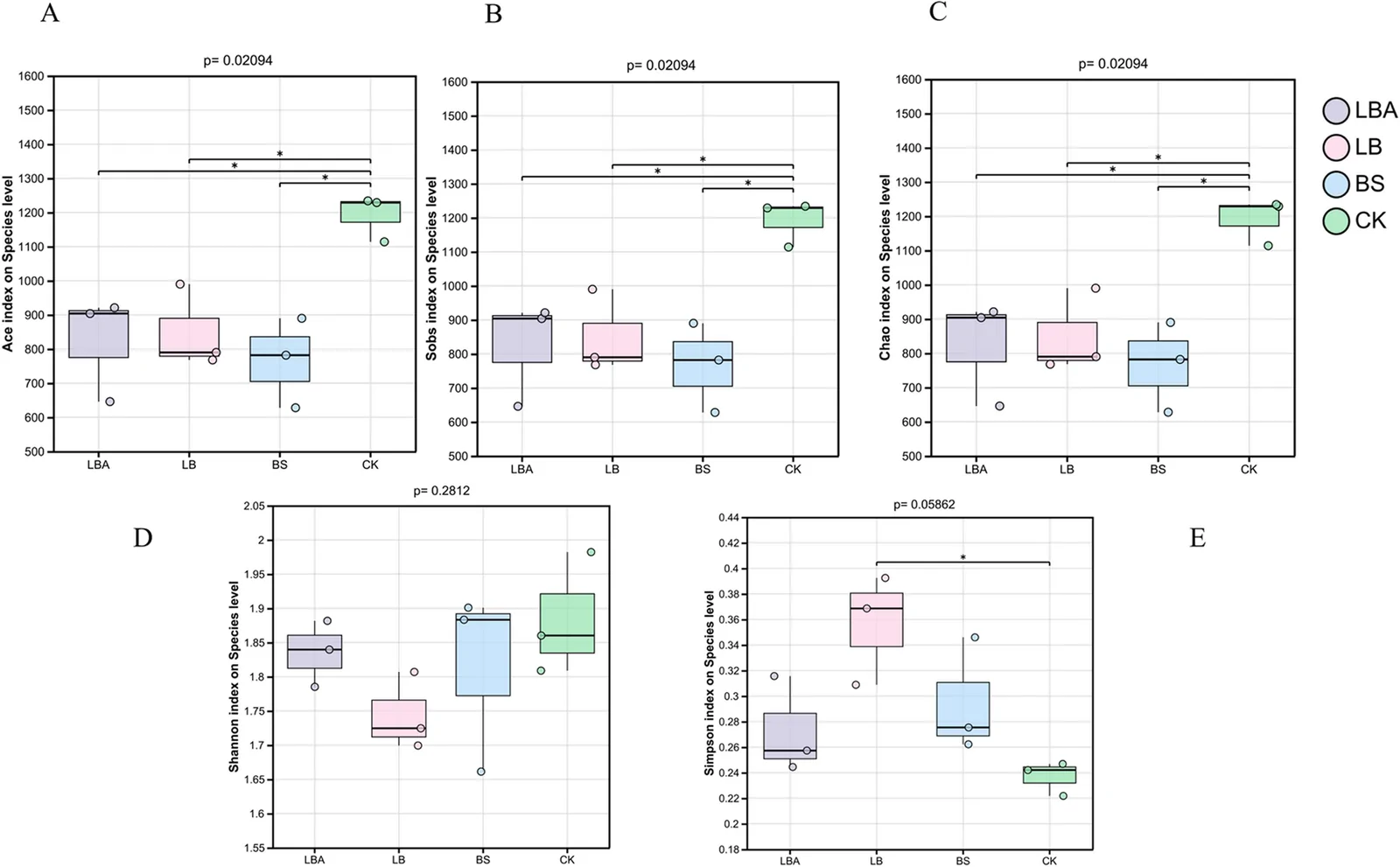
Effects of Composite Fermentation Microbial Communities on the Nutritional Quality of Caragana korshinskii Silage. **A** Ace index. **B** Sobs index.**C** Chao1 index. **D** Shannon index. **E** Simpson index. LBA, (*L. plantarum* + *B. subtilis* + *A. niger*), LB, (*L. plantarum*), BS, (*B. subtilis*), and CK, (control)

It can be seen from Fig. 2 that microbial additives can significantly affect the microbial community of *Caragana korshinskii* silage after 30 days of silage. The LBA treatment showed higher Chao1 and ACE values than the other inoculated groups, indicating that this consortium preserved a relatively greater taxonomic richness, including low-abundance members. However, its lower Simpson index, together with the genus-level composition, suggests that the community became more strongly dominated by a limited number of taxa, particularly *Lactobacillus*. The Shannon index of the LBA group was higher than those of the LB and BS groups but lower than that of the CK group, indicating that LBA maintained more richness than the single-strain inoculations while still undergoing directional selection toward a less even, fermentation-adapted community. In contrast, the LB and BS treatments showed reduced richness and a more simplified bacterial structure relative to CK. These results indicate that the synthetic consortium did not simply increase overall diversity, but rather restructured the silage microbiota into a functionally enriched yet uneven community dominated by beneficial fermentative bacteria.

**Fig. 3:**
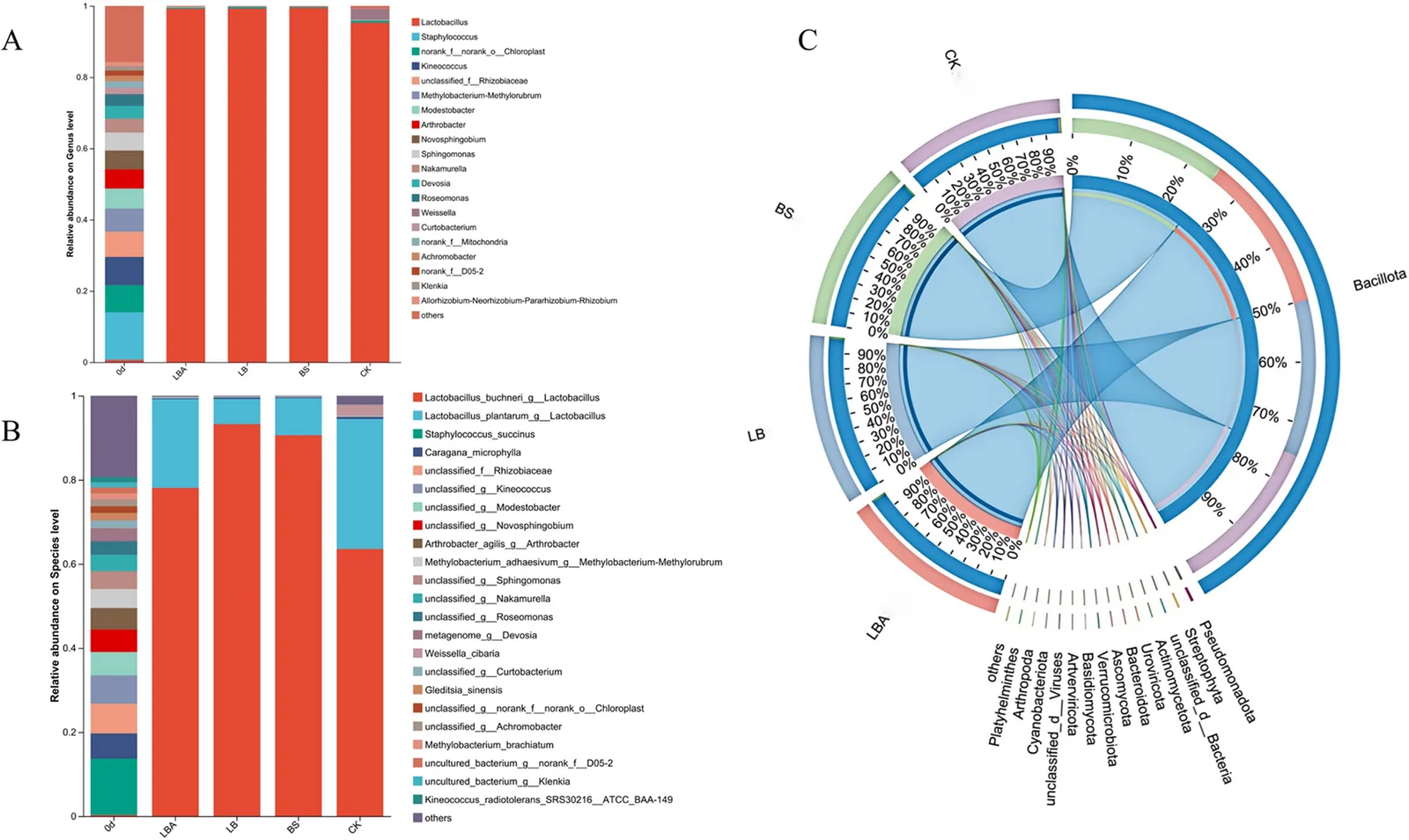
Dynamic changes in bacterial community composition of Caragana korshinskii Silage before and after ensiling under different microbial treatments. **A** The microbial composition of genus; (**B**) The microbial composition of species; (**C**) Circos diagram illustrating the distribution and contribution of major bacterial phyla across different treatments after 30 days of ensiling

In order to further understand the dynamic succession of alfalfa silage bacterial community under the addition of compound fermentation agent, we evaluated the bacterial community at the genus and species level (Fig. 3). At the genus level, *Staphylococcus* and *Kineococcus* accounted for 13.39% and 7.96%, followed by *Rhizobiaceae*, *Methylorubrum* and *Modestobacter* before ensiling. After 30 days of ensiling, the Caragana korshinskii silage flora was affected by the compound fermentation flora, and all treatment groups mainly contained *Lactobacillus* (95.30% − 99.50%). The LBA-30d, LB-30d, and BS-30d treatment groups exhibited largely similar bacterial compositions, primarily dominated by *Lactobacillus* and *Methylorubrum*. Pre-ensiling, the relative abundance of *Lactobacillus* was 1.53%. During the ensiling period, the abundance of *Lactobacillus* increased progressively, reaching 99.19% (LBA-30d), 99.27% (LB-30d), 99.50% (BS-30d), and 95.30% (CK-30d) at 30 d of ensiling. Conversely, the relative abundance of *Methylorubrum* exhibited a declining trend. Post-ensiling (at 30 d), the CK treatment group exhibited the highest relative abundance of *Weissella* at 3.06%. In contrast, the LBA, LB, and BS treatment groups showed increased relative abundances of *Lactobacillus* compared to the CK group, with *Weissella* undetected in these groups. Notably, the BS treatment group achieved a *Lactobacillus* abundance of 99.50%, surpassing all other treatments (Fig. 3A). At the species level, *Staphylococcus* (13.38%) and *Rhizobiaceae* (7.06%) were the main bacteria before silage, followed by *Kineococcus* and *Modestobacter* and *Novosphingobium*. After 30 days of ensiling, the Caragana korshinskii silage flora was affected by the compound fermentation flora. The bacterial species in the LBA-30d, LB-30d, and BS-30d treatment groups were basically similar, mainly containing *Lactobacillus buchneri* and *Lactobacillus plantarum*. The relative abundance of *Lactobacillus buchneri* was 5.32% before silage. With the increase of silage time, *Lactobacillus buchneri* showed an upward trend. The relative abundance of LBA-30d, LB-30d, BS-30d and CK-30d was 78.07%, 93.25%, 90.60% and 63.65%, respectively. *Lactobacillus plantarum* was not found before ensiling, and the relative abundance of *Lactobacillus plantarum* in all treatment groups after ensiling was 21.11%, 5.97%, 8.81% and 30.90%, respectively. After silage, the relative abundance of *Lactobacillus plantarum* in CK treatment group was higher than that in other treatments. The relative abundance of *Lactobacillus buchneri* in LBA, LB and BS treatment groups increased compared with CK treatment group, and *Weissella cibaria* was not found. The relative abundance of *Weissella cibaria* in CK treatment group was 2.54%, and the relative abundance of *Lactobacillus buchneri* in LB treatment group was 99.50%, which was higher than other treatments (Fig. 3B). The Circos diagram (Fig. 3C) further showed that the proportion of adaptive *Bacillota* at the phylum level in the BS/LB/LBA group reached 90%, which was consistent with the characteristics of the core flora of silage fermentation, indicating that lactic acid bacteria (mainly *Lactobacillus*) may become dominant bacteria, reflecting the function of the compound fermentation flora.

**Fig. 4:**
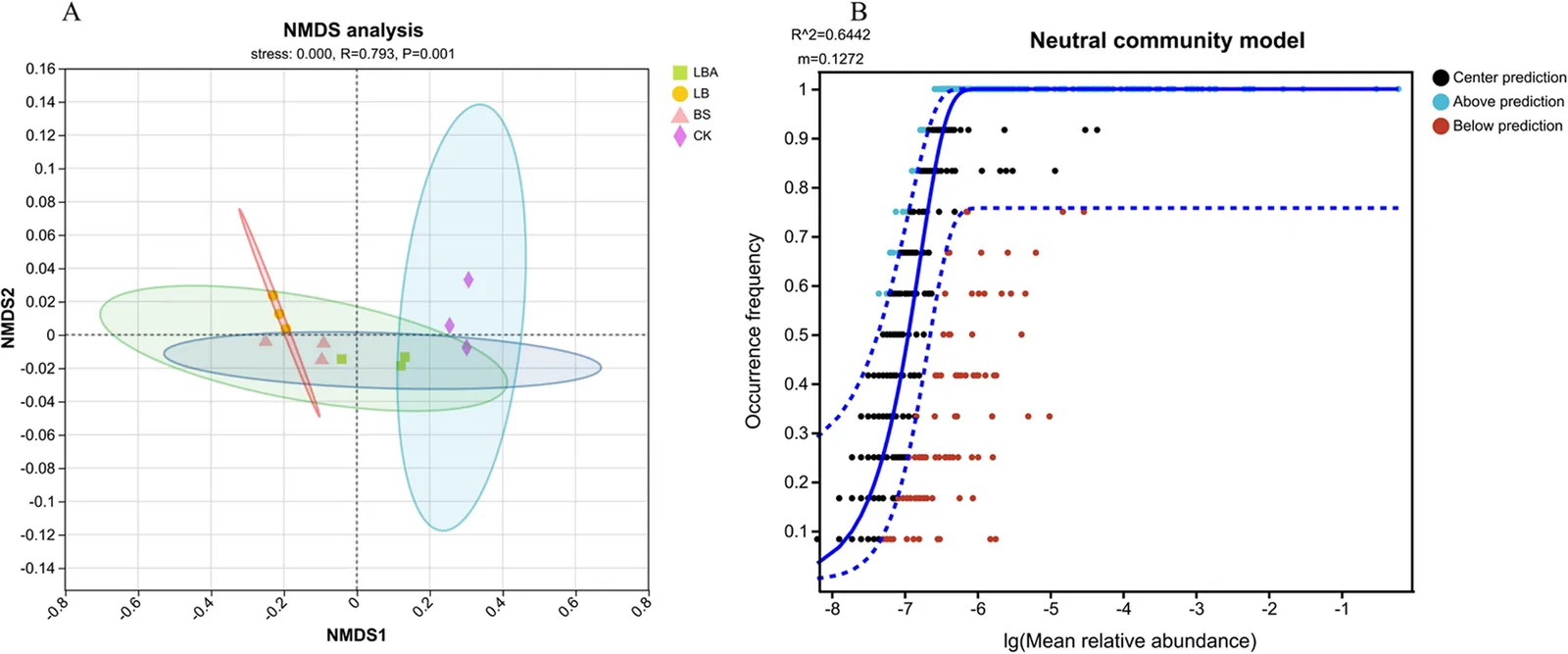
Beta-diversity analysis and community assembly mechanisms of bacterial communities in Caragana korshinskii Silage after 30 days of fermentation. **A** Non-metric multidimensional scaling (NMDS) analysis based on Bray–Curtis dissimilarity; (**B**) Neutral community model (NCM) analysis depicting the relationship between species occurrence frequency and relative abundance. LBA, *(L. plantarum* + *B. subtilis* + *A. niger*), LB, (*L. plantarum*), BS, (*B. subtilis*), and CK, (control)

The analysis of non-metric multidimensional scale (NMDS, Fig. 4A) and neutral community model (NCM, Fig. 4B) in this study revealed the changes of bacterial community during anaerobic fermentation. The stress value of 0.000 indicates that the data fit well with the model. NMDS analysis showed the similarities and differences between different groups of samples. The figure shows the relative position of the four treatment group samples in the figure. The CK treatment group was significantly different from other treatment groups, and the samples were distributed in the right area of the figure. LBA, LB and BS are alternately overlapped in the central region of the graph. With the support of the NCM model, NMDS analysis provided a clear insight into the changes in bacterial communities during anaerobic fermentation (4B). The results showed that the addition of microbial inoculants had a significant effect on the microbial composition of 30 d *Caragana korshinskii* silage. NMDS analysis revealed clear separation between the control group and inoculated treatments, indicating that microbial inoculation reshaped the community structure. NCM analysis suggested that the LBA community was more strongly governed by deterministic processes, reflecting the directional selection imposed by the synthetic consortium. These analyses indicate that the improved performance of LBA is associated with treatment-driven community assembly rather than stochastic variation.

**Fig. 5:**
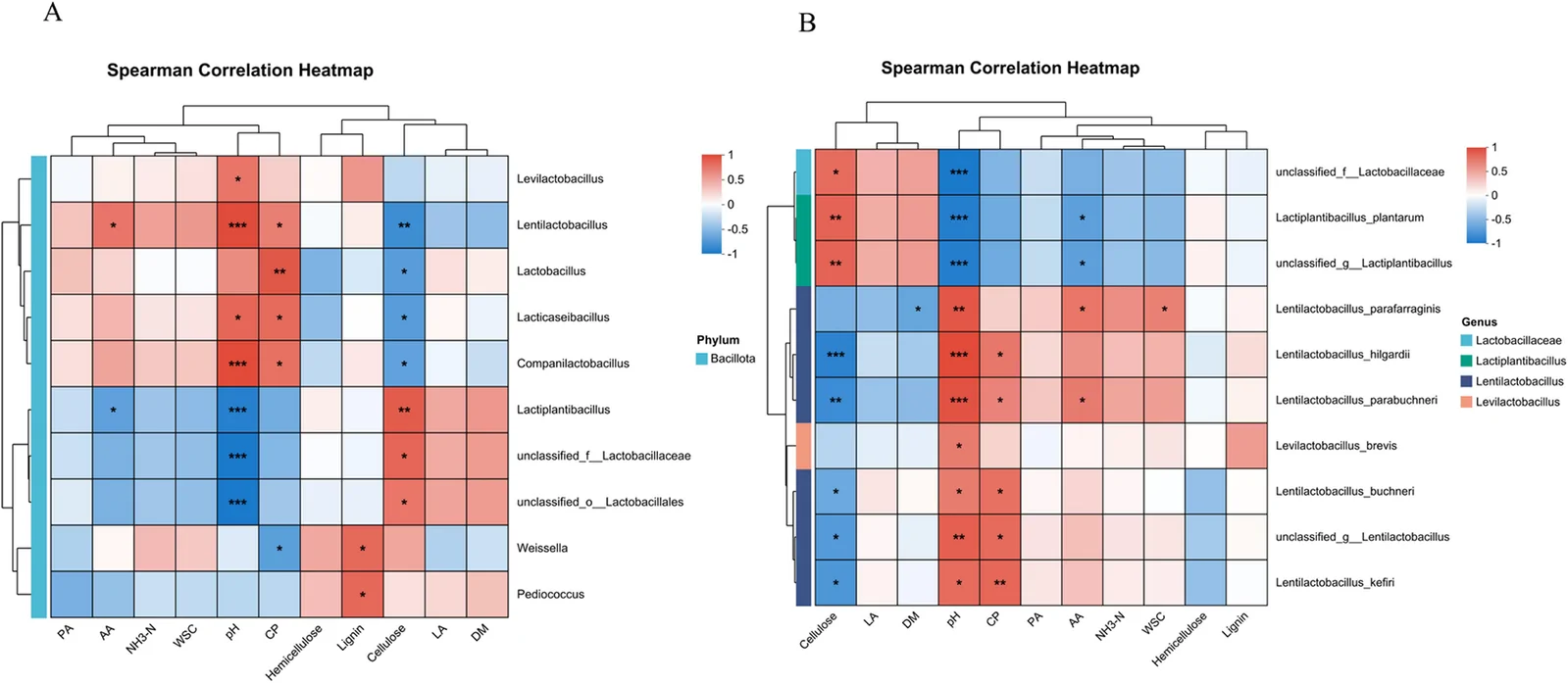
Spearman correlation analysis between microbial community composition and fermentation quality parameters of Caragana korshinskii Silage after 30 days of fermentation. **A** Correlation heatmap between dominant bacterial genera (top 10 in relative abundance) and silage fermentation characteristics. **B** Correlation heatmap between dominant bacterial species (top 10 in relative abundance) and silage fermentation characteristics. Color intensity represents the strength of correlation, with red indicating positive correlation and blue indicating negative correlation. Asterisks indicate significant correlations (*P* < 0.05)

Spearman heat map analysis visually showed the correlation between the microbial community (genus level) of *Caragana korshinskii* silage and the silage fermentation quality (Fig. 5A). The abscissa in the map was the microorganisms with abundance in the top 10 different genera, and the ordinate was different silage fermentation indicators. It can be seen from the map that the genus *Lentilactobacillus* is significantly positively correlated with the CP and AA content of *Caragana korshinskii* silage (*P* < 0.05); *Lactiplantibacillus* was significantly positively correlated with CP and pH content of *Caragana korshinskii* silage (*P* < 0.05). *Weissella* was significantly negatively correlated with CP content of *Caragana korshinskii* silage, but significantly positively correlated with lignin content (*P* < 0.05). *Lactobacillus* was significantly positively correlated with CP content (*P* < 0.05), and *Lacticaseibacillus* was significantly positively correlated with CP content and pH value (*P* < 0.05). *Companilactobacillus* was significantly positively correlated with the pH value of *Caragana korshinskii* silage (*P* < 0.01). *Lactiplantibacillus*, *Lactobacillaceae* and *Lactobacillales* were significantly negatively correlated with pH value (*P* < 0.01), and significantly positively correlated with hemicellulose (*P* < 0.05).

Correlation analysis between microbial community (species level) and silage fermentation quality of *Caragana korshinskii* silage. The abscissa in the diagram is the different microorganisms in the top 10 abundances, and the ordinate is different silage fermentation indicators (Fig. 5B). From the diagram, it can be seen that *Lentilactobacillus buchneri* and *Lentilactobacillus_kefiri* were significantly positively correlated with pH value and CP content of Caragana korshinskii silage, but negatively correlated with NDF content (*P* < 0.05). *Lentilactobacillus parabuchneri* was significantly positively correlated with CP and AA contents in *Caragana korshinskii* silage (*P* < 0.05). *Lactiplantibacillus plantarum*, *Lactiplantibacillus* and *Lactobacillaceae* were significantly negatively correlated with pH value of *Caragana korshinskii* silage (*P* < 0.01), but significantly positively correlated with cellulose content (*P* < 0.05). *Lentilactobacillus parafarraginis* was significantly positively correlated with pH value, AA and WSC content of *Caragana korshinskii* silage (*P* < 0.05), but significantly negatively correlated with DM content (*P* < 0.05). *Levilactobacillus brevis* was significantly positively correlated with the pH value of *Caragana korshinskii* silage (*P* < 0.05). *Lentilactobacillus hilgardii* and *Lentilactobacillus parabuchneri* were significantly positively correlated with pH value of *Caragana korshinskii* silage, and significantly positively correlated with cellulose content (*P* < 0.01).

### Effects of compound fermentation flora on microbial metabolic function of *Caragana korshinskii* silage

**Fig. 6:**
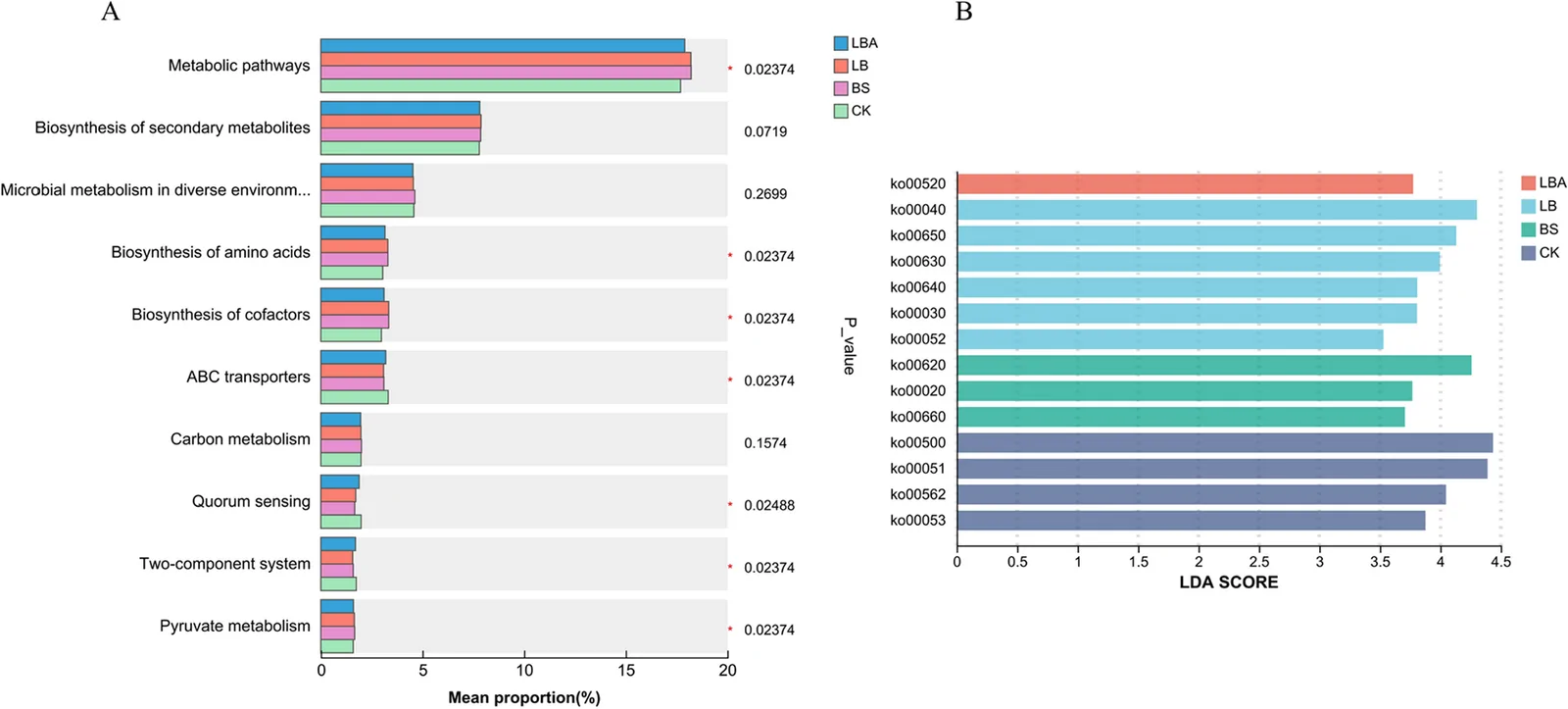
Predicted functional profiles of bacterial communities in Caragana korshinskii silage based on Tax4Fun analysis of 16 S rRNA gene sequencing data after 30 days of fermentation. **A** Relative abundance of major KEGG level-2 functional pathways among different treatments. **B** LEfSe analysis identifying significantly enriched KEGG pathways across treatments, with a linear discriminant analysis (LDA) score threshold of 2. LBA, (*L. plantarum* + *B. subtilis* + *A. niger*), LB, (*L. plantarum*), BS, (*B. subtilis*), and CK, (control)

To predict the functional potential of bacterial communities, we performed Tax4Fun analysis based on 16 S rRNA gene sequencing data. Comparative analysis of KEGG pathways found that (Fig. 6A), the abundance of 10 KEGG functions varied among different treatments, and the abundance of Metabolic pathways was the highest (*P* < 0.05). The abundance of Metabolic pathways in LBA treatment group was significantly higher than that in CK treatment group, but significantly lower than that in BS and LB treatment groups (*P* < 0.05). The abundance of Metabolic pathways and Biosynthesis of amino acids Pyruvate metabolism in BS treatment group was significantly higher than that in other treatment groups (*P* < 0.05). The abundance of ABC transporters, Quorum sensing and Two-component system in CK treatment group was significantly higher than that in LBA, LB and BS groups. These pathways are commonly associated with microbial stress response, interspecies competition, and environmental sensing, suggesting that the CK silage harbored a more dynamic and potentially competitive microbial community compared with the inoculated treatments. The overall KEGG functional profiles of the LB and BS treatment groups were relatively similar, however, LEfSe analysis identified differences in several specific predicted pathways between the two groups. Through the LEfSe multi-level functional difference discriminant analysis (LDA threshold of 2) of *Caragana korshinskii* silage microbial community, it was found that (Fig. 6B), there were 14 KEGG metabolic functions with significant differences in abundance between groups (*P* < 0.05). The significantly enriched KEGG pathways in the LBA treatment group were Amino sugar and nucleotide sugar metabolism; Pentose and glucuronate interconversions, But anoate metabolism and Glyoxylate and dicarboxylate metabolism were significantly enriched in LB treatment group. The significantly enriched KEGG pathways in the BS treatment group were pyruvate metabolism, the citrate cycle (TCA cycle), and C5-branched dibasic acid metabolism. The significantly enriched species in the CK treatment group were Starch and sucrose metabolism, Fructose and mannose metabolism and Inositol phosphate metabolism.

**Fig. 7:**
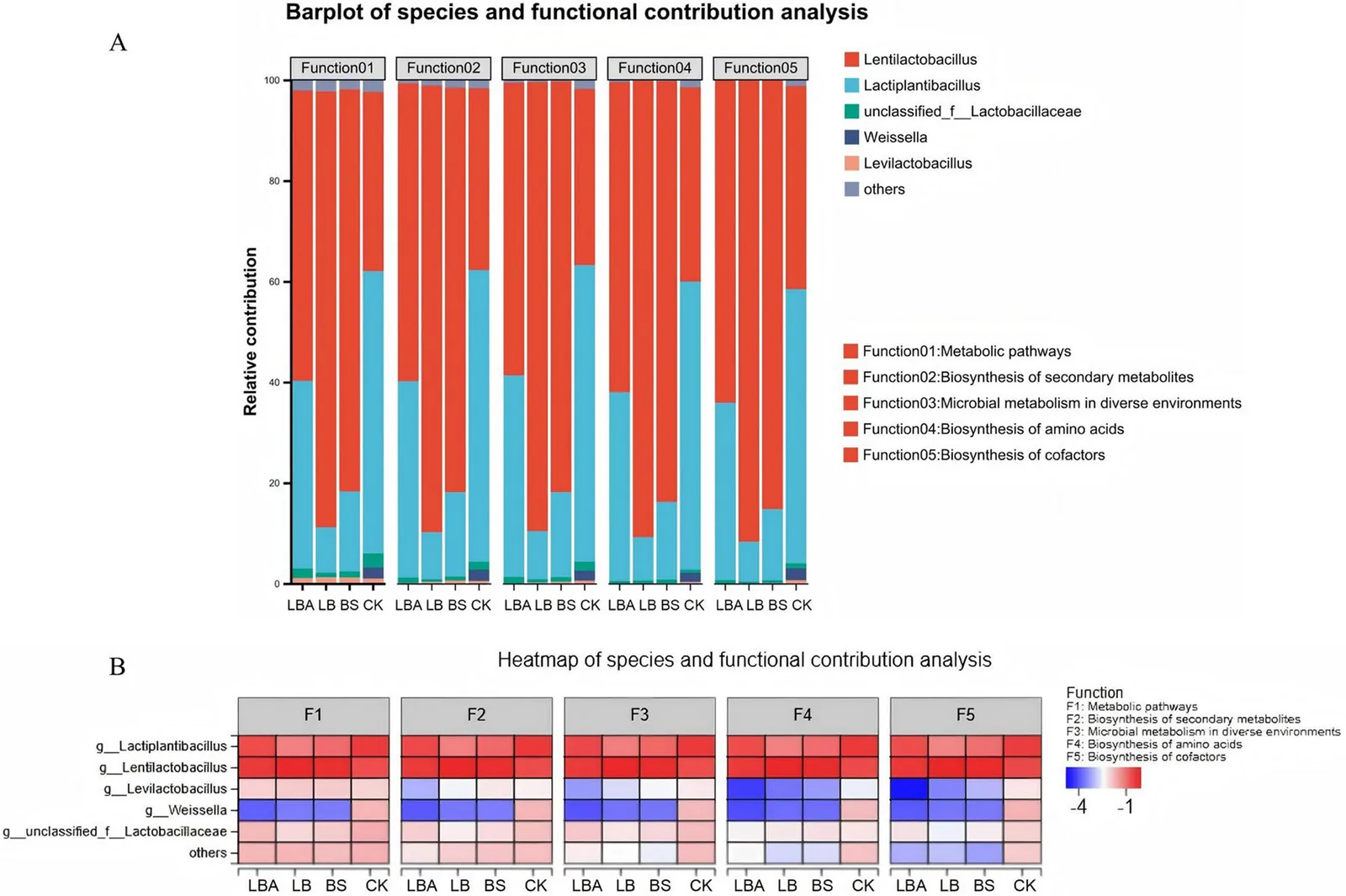
Contribution of dominant bacterial taxa to key metabolic functions in Caragana korshinskii Silage after 30 days of fermentation. **A** Species contribution analysis showing the relative contributions of major bacterial genera to selected KEGG metabolic pathways. **B** Heatmap illustrating correlations between dominant bacterial genera and key metabolic functions across treatments. Color gradients indicate the magnitude of contribution or correlation, with red representing higher values and blue representing lower values. LBA, (*L. plantarum* + *B. subtilis* + *A. niger*), LB, (*L. plantarum*), BS, (*B. subtilis*), and CK, (control)

According to the taxon–function contribution analysis (Fig. 7A), *Lentilactobacillus* and *Lactiplantibacillus* showed the highest contributions to the five selected KEGG pathways. *Lactiplantibacillus* was the core driving bacteria of silage fermentation, and its efficient glycolysis ability (such as decomposing soluble sugar in *Caragana korshinskii* to produce lactic acid) directly determined the pH decline rate at the initial stage of fermentation and inhibited the growth of spoilage bacteria. *Lactiplantibacillus* is a diversity of auxiliary metabolic branches (such as pentose and hexose metabolism), which may be involved in the decomposition of complex carbohydrates (lignin and cellulose degradation products in *Caragana korshinskii*) and broaden the scope of substrate utilization. The contribution of *Lentilactobacillus* in LB treatment group was significantly higher than that in other treatment groups, all above 80%, while that in CK treatment group was significantly lower than that in other treatment groups, but the contribution of *Lactiplantibacillus* was the highest (*P* < 0.05). Among the functions of Metabolic pathways, the contribution of *Lactiplantibacillus* in LB treatment group was significantly the lowest, accounting for 9.01%, while *Lactobacillaceae* had a contribution of 2.8% in CK treatment group, followed by LBA treatment group, in Biosynthesis of amino acids and Biosynthesis of cofactors. The contribution of *Lactobacillaceae* in LBA, LB and BS treatment groups was significantly the lowest (*P* < 0.05), but there was no significant difference among the three treatment groups. The species and functional contribution heat map analysis showed that (Fig. 7B), *Lentilactobacillus* and *Lactiplantibacillus* were significantly positively correlated with the five functions in all treatment groups (*P* < 0.05), indicating that exogenous addition of homologous lactic acid bacteria could strengthen its colonization advantage in silage, thereby enhancing the activity of metabolic pathways and promoting the rapid accumulation of lactic acid and efficient use of nitrogen. Furthermore, in the LBA, LB and BS treatment groups, *Weissella* was significantly negatively correlated with the five functions (*P* < 0.05), indicating that the addition of microbial agents inhibited the activity of proteolytic bacteria and reduced the production of adverse metabolites.

## Discussion

### Effect of composite fermentation flora on rumen fermentation characteristics of Caragana korshinskii silage in vitro

*Caragana korshinskii* with a high content of lignocellulose, when used as roughage for animals, has poor palatability and low digestion and utilization rate. At present, fungi can be used as additives to degrade fiber content and increase CP content. Abubakar et al. [22] showed that after adding fungi for solid-state fermentation to degrade wheat straw, the content of lignocellulose was significantly reduced, improving the palatability and digestibility of wheat straw silage feed. Fibrous feed can promote the peristalsis of microorganisms in the animal gastrointestinal tract, increase the amount of fiber-degrading bacteria in the intestinal tract, enrich the microbial flora in the gastrointestinal tract, and improve the animal’s digestion rate of fibrous feed [23]. Halidai et al. [24] found that the higher the CP content in the feed, the more conducive it is to the growth and reproduction of beneficial bacteria, thereby increasing the degradation rate of the feed in the rumen. In this study, the in vitro dry matter digestibility (IVDMD) and neutral detergent fiber degradation rate (IVNDFD) in the LBA and LB treatment groups were significantly increased. These results suggest that the composite fermentation microbial consortium primarily promotes partial degradation of lignocellulosic structures, as indicated by improved in vitro digestibility and enrichment of carbohydrate-active metabolic pathways, rather than causing substantial changes in overall chemical composition (e.g., crude protein, NDF, ADF, ADL). Therefore, in this study, the addition of compound fermentation flora had a positive impact on the digestibility of Caragana korshinskii silage. LBA improved digestibility suggests a possible role of Aspergillus niger in delignification, but no direct fungal metagenomic evidence was obtained, as sequencing focused on bacteria; thus, its role remains inferential and requires further validation.

In vitro gas production is an important indicator for evaluating the degree of feed fermentation, which can reflect the digestion level and rate of feed by ruminants. The fermentation gas production of rumen microorganisms is positively correlated with the feed degradation rate. The higher the fermentability of the feed, the stronger the microbial activity and the greater the gas production. In this study, at 14 days of silage, the cumulative total gas production within 48 h in the LBA treatment group was significantly higher than that in other treatment groups. This might be because the quality of Caragana korshinskii silage feed in the LBA treatment group was higher, and the NDF content was negatively correlated with the gas production [25]. However, at 30 days of silage, there was no significant difference in gas production among each group at 18–48 h. This might be because rumen microorganisms had differences in the degradation of DM in Caragana korshinskii silage treated with different complex fermentation bacterial communities, which was similar to the research results of Xiong et al. [26]. The results of this experiment indicated that the gas production rate of the LBA treatment group was significantly higher than that of the other groups, suggesting that its fermentable substrates were more readily utilized by rumen microorganisms.

### Effects of complex fermentation flora on the microbial community and metabolic functions of Caragana korshinskii silage

This study showed that ensiling strongly shaped the microbial community structure of *Caragana silage*. After 30 days of ensiling, *Lactobacillus* became the absolute dominant genus in all groups, with a relative abundance of 95.30%–99.50%. This dominance should be regarded as a general outcome of successful ensiling rather than a unique effect of the composite microbial consortium. However, differences remained among treatments in community distribution, species-level composition, and minor taxa, particularly the enrichment of *Lactobacillus buchneri* and *L. plantarum*. This result is consistent with the results of NMDS and neutral community model (NCM) analyses. The NMDS analysis shows that after 30 days of fermentation, there are differences in the distribution of bacterial communities among different treatment groups. The control group samples tend to be separated from the inoculated groups, while the LBA, LB, and BS groups are relatively concentrated in the central area of the ordination diagram. This indicates that the addition of exogenous lactic acid bacteria can quickly occupy ecological niches and inhibit the growth of potential spoilage bacteria such as *Staphylococcus* and *Methylorubrum* [27]. That is, the microbial community structure of the compound bacterial agent treatment group (LBA, LB, BS) was significantly separated from that of the control group (CK), and the α diversity of the bacterial community decreased, further confirming the competitive advantage of lactic acid bacteria in the silage system [28]. It is worth noting that the abundance of *Weissella* was relatively high (3.06%) in the control group, while it was almost undetectable in the compound bacterial agent treatment group. Based on Spearman correlation analysis, *Weissella* was positively correlated with lignin accumulation. This suggests that the decrease of *Weissella* in the inoculated treatments may be associated with changes in microbial metabolites potentially affecting lignin degradation, although direct measurements of antibacterial metabolites were not performed [29]. In contrast, the significant positive correlation (*P* < 0.05) between *Lactobacillus* and crude protein (CP) indicates that the compound bacterial agent, by optimizing the fermentation environment, not only promotes lactic acid accumulation but also may reduce protein degradation, thereby maintaining the nutritional value of silage. Because species-level assignments from 16 S rRNA data are not definitive, our interpretations focus on genus-level patterns unless otherwise noted.

From the perspective of metabolic function, KEGG and LEfSe analyses revealed the significant impact of the compound bacterial agent on the functional pathways of microorganisms. The significant enrichment of glucose metabolism pathways (pyruvate metabolism, amino acid biosynthesis) in treatment groups (such as LB and BS) indicates that exogenous lactic acid bacteria accelerate the conversion of WSC to lactic acid by enhancing the efficiency of glycolysis, thereby reducing the pH value (the pH of the LBA treatment group was the lowest) [30]. The higher abundance of ABC transporters, quorum sensing, and two-component systems in the CK group further suggests a microbial community under environmental stress and competitive pressure, in contrast to the more stable, inoculant-dominated silage communities. It is worth noting that the taxon–function contribution analysis showed that *Lactiplantibacillus* and *Lentilactobacillus* were the core functional drivers. *Lactiplantibacillus* may contribute to rapid acidification through efficient carbohydrate fermentation [31], whereas *Lentilactobacillus* may be associated with carbohydrate utilization and fermentation-related metabolic pathways, thereby broadening substrate utilization potential [32]. Furthermore, the negative correlation between *Weissella* and metabolic function in the microbial agent treatment group further confirmed that the compound microbial agent optimized the quality of silage by inhibiting the activity of proteolytic bacteria and reducing the generation of adverse metabolites such as NH_3_-N [33]. All species-level claims in this study should be considered putative, and genus-level interpretations are more robust. Future studies employing shotgun metagenomic sequencing with species-resolved binning or metatranscriptomics are needed to confirm species-specific roles. In conclusion, the complex fermentation microbial community, by regulating the structure and metabolic function of the microbial community, collaboratively enhances the fermentation efficiency and nutritional value of Caragana korshinskii silage, providing a theoretical basis and technical support for the silage treatment of high-wood fiber raw materials.

## Conclusion

This study suggests that a synthetic cross-kingdom microbial community has the potential to improve the utilization of *Caragana korshinskii* silage and may help alleviate the limitations associated with its lignocellulosic structure. The synthetic microbial community composed of *Lactobacillus plantarum*, *Bacillus subtilis*, and *Aspergillus niger* was associated with improved fermentation quality, changes in microbial community structure, and higher in vitro digestibility of *Caragana korshinskii* silage. After ensiling, the relative abundance of *Lactobacillus* increased markedly in all groups, while the inoculated treatments showed differences in community composition compared with the control, accompanied by lower relative abundances of undesirable taxa such as *Staphylococcus* and *Weissella*. The LBA treatment showed the highest in vitro dry matter digestibility (49.68%), indicating a potential association between inoculation and improved nutritional performance. Overall, these findings suggest that synthetic microbial communities may represent a promising strategy for improving the feed utilization of high-fiber shrub resources and may provide useful guidance for forage development in arid agro-ecosystems.

## Supplementary Information

Supplementary Material 1.

## Data Availability

The datasets presented in this study can be found in online repositories. We uploaded the sequences data in the NCBI and got an accession number PRJNA1015568.
